# Targeting mRNA for Alzheimer's and Related Dementias

**DOI:** 10.1155/2014/757549

**Published:** 2014-04-27

**Authors:** Michael S. Wolfe

**Affiliations:** Brigham and Women's Hospital, Harvard Medical School, 77 Avenue Louis Pasteur, H.I.M. 754, Boston, MA 02115, USA

## Abstract

Brain deposition of the amyloid beta-protein (A**β**) and tau are characteristic features in Alzheimer's disease (AD). Mutations in the A**β** precursor protein (APP) and a protease involved in A**β** production from APP strongly argue for a pathogenic role of A**β** in AD, while mutations in tau are associated with related disorders collectively called frontotemporal lobar degeneration (FTLD). Despite intense effort, therapeutic strategies that target A**β** or tau have not yet yielded medications, suggesting that alternative approaches should be pursued. In recent years, our laboratory has studied the role of mRNA in AD and FTLD, specifically those encoding tau and the A**β**-producing protease BACE1. As many FTLD-causing tau mutations destabilize a hairpin structure that regulates RNA splicing, we have targeted this structure with small molecules, antisense oligonucleotides, and small molecule-antisense conjugates. We have also discovered that microRNA interaction with the 3′-untranslated region of tau regulates tau expression. Regarding BACE1, we found that alternative splicing leads to inactive splice isoforms and antisense oligonucleotides shift splicing toward these inactive isoforms to decrease A**β** production. In addition, a G-quadruplex structure in the BACE1 mRNA plays a role in splice regulation. The prospects for targeting tau and BACE1 mRNAs as therapeutic strategies will be discussed.

## 1. Introduction

### 1.1. Protein Aggregation in Alzheimer's and Related Dementias

Aberrant protein deposition is a common characteristic of neurodegenerative diseases, including Alzheimer's disease (AD), frontotemporal lobar degeneration (FTLD), Parkinson's disease, amyotrophic lateral sclerosis, and prion diseases [1]. In AD, two pathological features define the disease: amyloid plaques and neurofibrillary tangles [2]. Amyloid plaques are extraneuronal deposits primarily composed of the amyloid *β*-protein (A*β*), while neurofibrillary tangles are intraneuronal filaments composed of the otherwise microtubule-associated protein tau. These two pathological deposits were first described over a century ago by Alois Alzheimer himself in characterizing the disease that bears his name. The question of whether either of these proteinaceous deposits is involved in disease etiology or coincidental markers has been much debated and still remains unresolved.

While the role of the deposits* per se* in the pathogenesis of AD is unclear, evidence strongly implicates the A*β* and tau proteins as key components in the neurodegenerative pathway(s). The A*β* precursor protein (APP) undergoes sequential proteolysis by *β*-secretase and *γ*-secretase to produce A*β*, a 4 kDa amphipathic protein prone to aggregation [3]. Dominant missense mutations in and around the A*β* region of APP or in the catalytic component of *γ*-secretase (presenilin) are associated with early onset (<age 60 years) AD, and these mutations change the type or amount of A*β* to increase its tendency to aggregate. In recent years, considerable evidence has supported the hypothesis that soluble oligomeric forms of A*β* are particularly responsible for inhibiting proper synapse function and are toxic to neurons, although this hypothesis is still controversial [4]. While the amyloid plaques appear to be less detrimental, they may serve as a reservoir for soluble A*β* oligomers [5].

**Figure 1:**
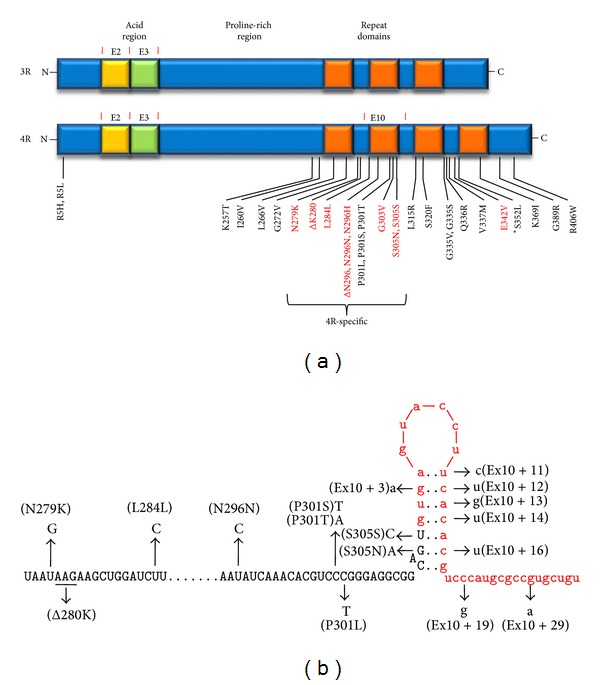
Tau splice isoforms, mutations, and splicing. (a) Alternative splicing of exon 10 results in tau isoforms with either 3 or 4 microtubule-binding repeat domains (3R or 4R tau). Alternative splicing of exons 2 and 3 is not shown. Site of FTLD-associated exonic mutations is indicated. Some of these mutations are silent and/or alter exon 10 splicing (red). Some of these mutations are also specific for the 4R isoforms of tau (bracket). (b) Stem-loop structure at the junction between exon 10 and intron 10. Site of FTLD-associated mutations in this structure destabilizing the stem-loop, increasing access to splicing factors and exon 10 inclusion, and resulting in increased 4R over 3R tau isoforms.

Tau is a 50–70 kDa microtubule-associated protein found in high levels in neurons, particularly in axons, and appears to function in microtubule formation, stability, and dynamics [6, 7]. The C-terminal region of tau is composed of 3 or 4 imperfectly repeated microtubule binding domains (Figure 1), but regions outside the repeat domains are also involved in microtubule binding [8–10]. In AD, tau becomes dissociated from microtubules, mislocalizes to neuronal cell bodies and dendrites, becomes hyperphosphorylated, and assembles into filaments [11]. These filaments comprise the neurofibrillary tangles described by Alzheimer that appear darkly upon silver staining. Genetic evidence in animals supports an essential role of tau in the A*β*-driven dementia of AD: knockout of the tau gene rescues learning and memory deficits in APP transgenic AD mouse models [12], a result that suggests that reduction of tau protein may be therapeutic for AD. Although mutations in tau are not associated with AD, tau-positive neurofibrillary tangles spatially correlate better with neurodegeneration than does A*β*, again suggesting that aberrant tau is downstream of A*β* and more proximal to neuronal cell death. In recent years, gathering evidence supports a model in which pathological tau is transmitted synaptically from neuron to neuron [13–15].

FTLD refers to the pathological situation in which the frontal and temporal lobes of the brain degenerate [16, 17]. In this pathology, different protein inclusions can be observed, including TAR DNA-binding protein 43 (TDP-43), fused-in-sarcoma (FUS), and tau. Clinically, these pathologies may manifest as Pick's disease, progressive nuclear palsy (PSP), corticobasal degeneration (CBD), argyrophilic grain disease (AGD), tangle-only dementia, and frontotemporal dementia with Parkinsonism linked to chromosome 17 (FTD-17). Dominant mutations in tau cause FTLD [18–20], not AD, but the presence of similar tau pathology in this subtype of FTLD (FTLD-tau) suggests that aberrant tau is also pathogenic in AD and that a variety of neuronal insults, including assembled forms of A*β*, can trigger changes in tau that ultimately lead to pathological deposition. FTLD mutations in tau result in microtubule dissociation, hyperphosphorylation, neuronal mislocalization, filament formation, and neurofibrillary tangles [16], the same molecular events that occur in AD. Thus, understanding the role of aberrant tau in FTLD-tau pathogenesis should be highly relevant to the role of tau in AD. Taken together, evidence strongly suggests that lowering tau levels is a worthwhile therapeutic goal for both AD and FTLD-tau. For AD, while targeting A*β* is expected to at least prevent disease onset, if not progression, targeting tau is more likely to slow or stop disease progression.

### 1.2. Targeting mRNA as an Alternative Therapeutic Strategy

A variety of approaches have been taken toward targeting the A*β* and tau proteins over the years. For A*β*, perhaps the leading strategy has been the inhibition of the proteases responsible for its production from APP, *β*-secretase (also called *β*-site APP cleaving enzyme 1 or BACE1), a membrane-tethered aspartyl protease related to pepsin [21], and *γ*-secretase, a membrane-embedded protease complex [22]. Much previous experience in the pharmaceutical industry with structure-based design of aspartyl protease inhibitors as drugs (e.g., HIV protease inhibitors, renin inhibitors) developing BACE1 inhibitors with appropriate CNS drug-like properties has been difficult [23], due to the long, shallow nature of the enzyme's active site and the tendency of many BACE1 inhibitors to be exported from the brain by P-glycoprotein. While these hurdles are being slowly overcome and several BACE1 inhibitors are in clinical trials, there is still enough concern to warrant consideration of alternative approaches to targeting BACE1.

*γ*-Secretase is also an aspartyl protease, although it is a complex of four integral membrane proteins and completely unrelated to soluble or membrane-tethered proteases [24]. Nevertheless, inhibitors of *γ*-secretase were more easily identified and several of these advanced into the clinic much more quickly than inhibitors of BACE1. However, *γ*-secretase cleaves other proteins besides APP, and some of these serve essential cell signaling roles in human biology [25]. For this reason, *γ*-secretase inhibitors have displayed mechanism-based toxicities that preclude approval for use in AD [26]. Currently, modulation of *γ*-secretase activity to reduce forms of A*β* that are particularly aggregation prone is the leading strategy for targeting this enzyme [26]. Another major approach to target A*β* is immunotherapy with anti-A*β* antibodies [27]. To some degree, these antibodies can access the brain and clear out neurotoxic A*β*. Although several of these have advanced through clinical trials, efficacy has been equivocal at best [28, 29].

Toward targeting tau, the major approach has been to develop inhibitors of kinases thought to be important in forming the hyperphosphorylated forms of the protein associated with tau filaments and neurofibrillary tangles [30]. While tau is phosphorylated at many of its Ser, Thr, and Tyr residues and a variety of kinases have been implicated in phosphorylating these sites, several sites are particularly associated with pathological tau, and certain kinases are reasonable candidates for phosphorylating these specific sites [31]. These include CDK5, GSK3, and MARK. While inhibitors of these kinases have been developed, potency, specificity, and brain penetration are often issues and few if any have advanced into clinical trials. Moreover, questions remain about the importance of any particular kinase, or even if tau hyperphosphorylation is pathogenic or merely coincidental, raising concerns that improving the properties of the drugs may not result in efficacy.

Thus to date all clinical trials for AD therapeutics have failed to progress to approved drugs. While the reasons for these failures vary depending on the specific agent and the approach, collectively these discouraging results suggest that alternative strategies should be pursued, at least in parallel. Toward that end, in recent years our laboratory has focused on mRNA relevant to AD and related dementias as potential targets. This approach seems particularly appropriate in cases where the protein target is not considered “druggable,” with tau being a prime example. In general, the mRNA encoding the pathogenic protein or enzyme can be targeted so as to shift alternative splicing toward less toxic or inactive forms or knocked down to simply reduce all forms of the protein. Targeting the mRNA may be accomplished with small molecules (where structure is present in the relevant region of the mRNA), with antisense oligonucleotides (ASOs), with small interfering RNA (siRNA), or with microRNA (miRNA). For AD and FTLD-tau, we have focused on mRNA encoding tau and BACE1 as potential targets and described these approaches below.

## 2. Tau mRNA

### 2.1. Disease-Causing Mutations Alter Tau mRNA Splicing

Nearly 40 mutations associated with FTLD-tau have been identified [32]. These are all either missense mutations, mutations that affect splicing, or both, and almost all cluster in the portion encoding the C-terminal region or in an intervening sequence near exon 10 (Figure 1(a)). The C-terminal missense mutations all appear to impair tau binding to microtubules and the ability of tau to promote microtubule assembly. Most of the silent mutations increase the 4R-to-3R ratio by modulating alternative splicing of exon 10. Missense mutations found within exon 10 only affect the 4R isoforms (Figure 1), while those found outside this region affect all six tau isoforms. Along with the consistent tau deposition in these familial cases and the observation that complete knockout of tau in mice does not lead to neurodegeneration, the fact that all but one of the disease-associated mutations [33] are dominant strongly suggests a gain of a toxic function: one normal copy remains, and, in the case of exon 10 missense mutations, even the 3R tau isoforms translated from the disease allele are normal. No disease-associated mutations have been identified that lead to either a truncated protein or the nonsense-mediated decay of message.

Most of the silent and intronic mutations near the exon 10 5′ splice site enhance exon 10 inclusion and destabilize a stem-loop structure at the exon-intron junction (Figure 1(b)). The stem-loop structure was hypothesized [18, 19, 34] upon the initial discovery of FTLD-tau mutations in the tau gene, noting the apparent self-complementarity in this region. Subsequent determination of the solution structure of an oligonucleotide based on this exon-intron junction by nuclear magnetic resonance spectroscopy [35] led to refinement of the stem-loop model to seven specific base pairs (Figure 1(b)) with an adenosine bulge between the sixth and seventh base pair. The structure further shows that this unpaired purine ring is intercalated back into the A-form RNA duplex. Disease-associated mutations found in this region would be predicted to destabilize the stem-loop and make this site more available to splicing factors (specifically the U1 snRNP that interacts with the 5′ splice site). Thermal stability studies of oligonucleotides demonstrated that disease-associated mutations within the putative stem-loop lower the melting temperature of the RNA duplex (i.e., where the double-stranded RNA dissociates with the single-stranded form) [35, 36]. A minigene construct encoding exons 9–11 recapitulates normal tau exon 10 splicing for the wild-type sequence and increased exon 10 inclusion for disease-causing mutations [37]. This minigene has been used in our lab to demonstrate that other mutations specifically designed to enhance stability of the stem-loop (and located distal to the U1 snRNP binding site) reduce exon 10 inclusion to decrease 4R/3R as predicted [36], validating the stem-loop as a* bona fide* structure involved in the regulation of tau exon 10 splicing and worthy of consideration as a therapeutic target.

### 2.2. Targeting Tau Exon 10 Splicing with Small Molecules [38–40]

Having validated the tau stem-loop RNA as a significant regulatory element in controlling tau mRNA splicing, we designed a high-throughput screen to identify small molecule ligands of the stem-loop RNA and developed other assays to validate the results of this screen [38]. Such compounds should stabilize the stem-loop and lower the 4R-to-3R ratio (i.e., do the opposite of disease-causing mutations). This approach may lead to new therapeutic agents for not only familial FTLD-tau with tau mutations but also all forms of FTLD-tau in which 4R tau isoforms predominate in neurofibrillary tangles (e.g., PSP and CBD) [16, 41]. Moreover, the possibility remains that shifting the balance of 4R/3R tau in AD may be beneficial as well. Although evidence for changes in tau RNA splicing in AD has been equivocal [42, 43], the 4R isoforms may be more prone to aggregate and seed coaggregation with 3R tau isoforms, as the repeat domains can transition from random coil to *β*-sheet conformations and are sufficient for filament formation [44].

**Figure 2:**
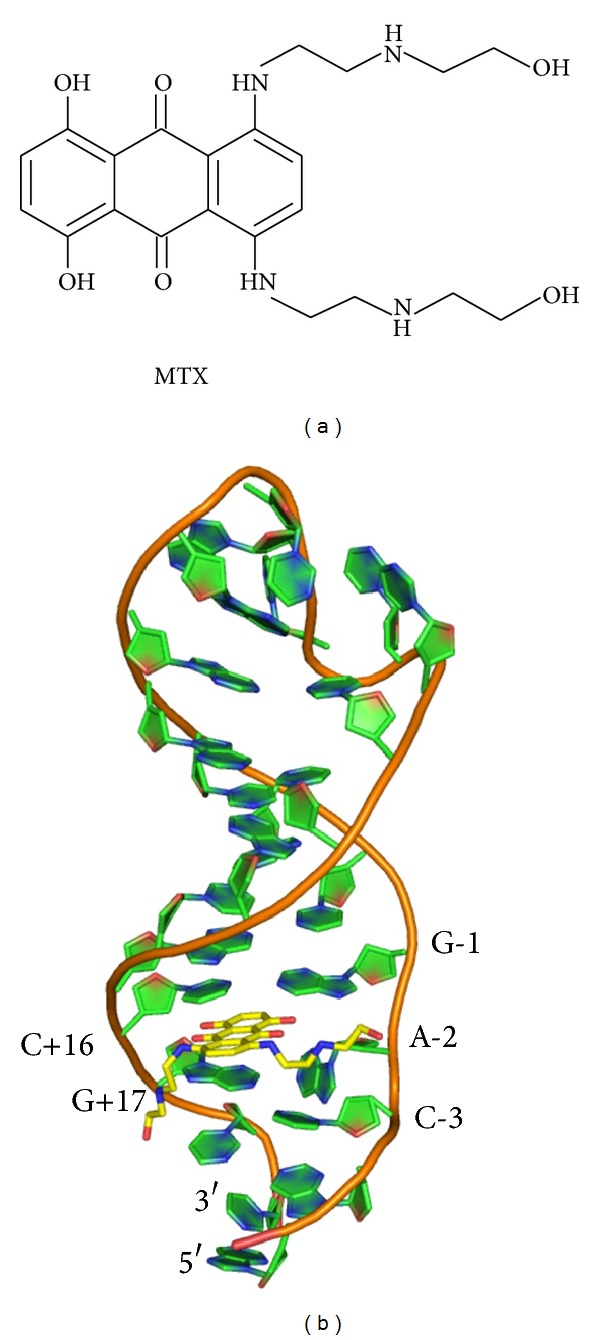
(a) Mitoxantrone structure (MTX). (b) NMR solution structure of MTX (yellow) bound to the tau RNA stem-loop. The aromatic region is intercalated between two G-C base pairs and flanking an unpaired adenosine. Side chains lie along the major groove.

Aminoglycosides are known to bind to double-helical RNA structures, including an oligonucleotide representing the tau mRNA stem-loop [45]. Based on the NMR-determined structure of neomycin bound to the tau stem-loop [45], we designed and synthesized pyrene-conjugated aminoglycosides as fluorescent probes for screening. Binding of the pyrene-aminoglycoside to the stem-loop quenches the pyrene fluorescence through intercalation. Thus, compounds that compete with such probes for stem-loop binding would increase levels of unbound probe and increase fluorescence. This approach was first validated with unconjugated aminoglycosides, demonstrating concentration-dependent increases in fluorescence, and then miniaturized for automated screening of 130,000 drug-like compounds. Though a number of compounds were identified as possible hits in this assay, only one passed secondary and tertiary biochemical tests: the anticancer drug mitoxantrone (MTX, Figure 2).

**Figure 3:**
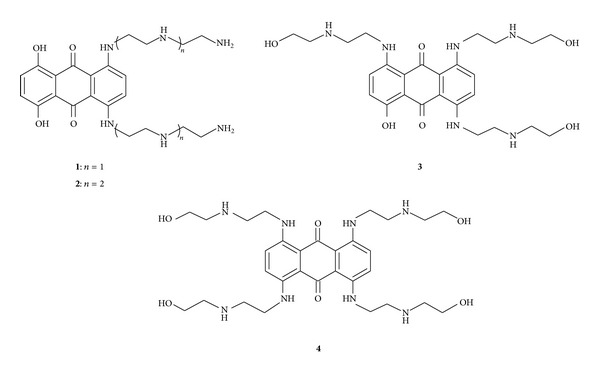
MTX analogs that retain or enhance the ability to bind and stabilize the tau RNA stem-loop structure.

The NMR solution structure of the tau mRNA-MTX complex [39] revealed that MTX binds in a region near the base of the stem, at the site of the unpaired adenosine (Figure 2). The aromatic ring system of the compound intercalates between two stacked G-C base pairs, and the side chains of the compound bind in the major groove via a combination of hydrogen bonding and electrostatic interactions. We then designed and synthesized a set of MTX analogues to explore structure-activity relationships and test the validity of the NMR-derived MTX-RNA 3-D structure [40]. We determined that the aromatic core and both side chains are required for RNA binding comparable to MTX; neither the side chain alone, the aromatic core alone, nor the aromatic core with only one side chain bind well, if at all. However, conversion of one or two of the terminal side chain hydroxyls to amine (e.g.,** 1**, Figure 3) led to a substantial increase in binding and stabilization, and extension with an additional ethylamine (e.g.,** 2**, Figure 3) gave comparable activities. Moreover, additional side chains (tri- and tetra-substituted** 3** and** 4**, Figure 3) were well tolerated, giving potencies comparable to MTX. These results and those with other MTX analogs were all compatible with the NMR model of MTX binding to tau RNA.

### 2.3. Targeting Tau Exon 10 Splicing with Bipartite Antisense Oligonucleotides [46]

**Figure 4:**
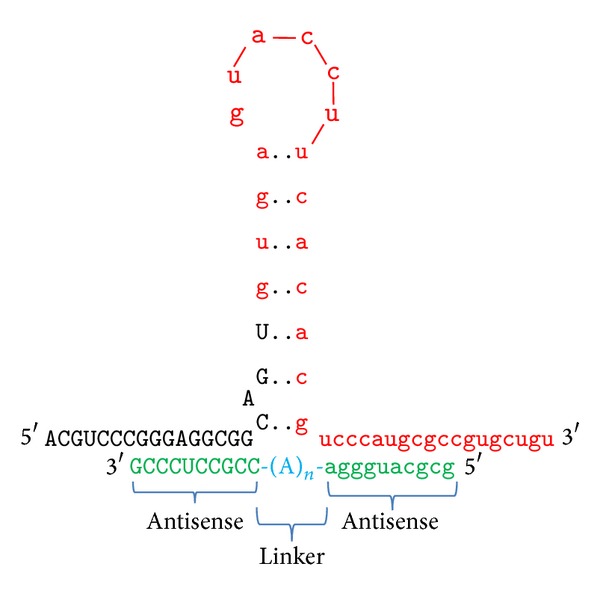
Design of bipartite antisense oligonucleotides targeting the tau RNA stem-loop structure.

In a complementary strategy to target the tau hairpin structure, we designed bipartite antisense oligonucleotides (ASOs), composed of two antisense regions within a single oligonucleotide, with spacer adenosine nucleotides [46]. Our designed bipartite ASOs have up to 10 bases on either side, complementary to their respective partner regions that flank the stem-loop, and have 0, 1, 2, or 3 adenosine spacers (Figure 4). The spacer adenosines are to bridge any potential gap between the flanking sequences due to the presence of the stem. Adenosine was chosen because there are no complementary uridines at the base of the stem.

We found that such bipartite ASOs do indeed bind to the tau pre-mRNA in the regions flanking the stem-loop. Electrophoretic mobility shift assays (EMSAs) demonstrated binding of bipartite DNA ASOs comparable to (or even better than) a control ASO targeting a linear sequence at the exon-intron boundary. DNA ASOs shorter than 7 nucleotides on either side of the linker did not bind, while 8 nucleotides on either side were sufficient for substantial binding. RNase protection assays demonstrated that both flanking regions were occupied by the designed ASOs. Bipartite RNA ASO counterparts with 2′-*O*-methylribose and phosphorothioate bonds (providing stability in cells) showed similar effects in EMSA but could also decrease 4R tau mRNA and increase the 3R tau form from either a tau minigene in SK-N-SH neuroblastoma cells or endogenous tau in HEK293 cells, with no effect on overall tau mRNA levels.

**Figure 5:**
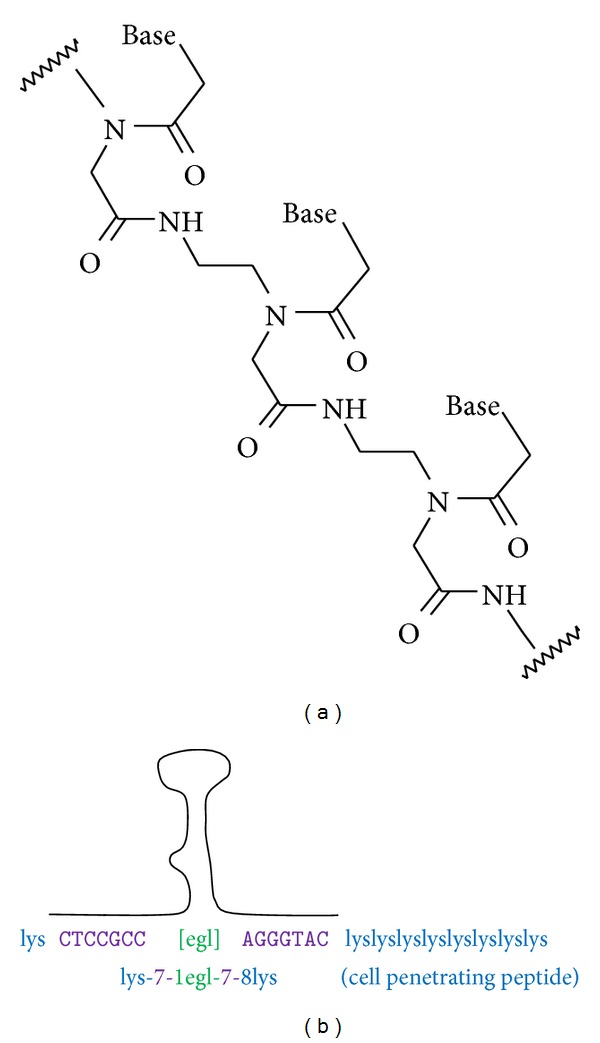
Bipartite peptide-nucleic acids (PNAs) and tripartite MTX-PNA. (a) General structure of PNA. (b) Designed bipartite PNA targeting the tau RNA stem-loop structure. Antisense PNA regions in purple; ethylene glycol linker (egl) in green; lysines added for solubility and cell penetration in blue.

In addition, we synthesized and tested bipartite peptide-nucleic acid (PNA) oligos. PNA contains a peptide backbone with nucleobase substituents, chemically and metabolically stable oligos that effectively target complementary RNA sequences and act as antisense molecules (Figure 5(a)) [47]. The design of PNA molecules targeted to the tau stem-loop flanking regions is shown in Figure 5(b). Two complementary PNA sequences were linked via 8-amino-3,6-dioxaoctanoic acid (egl) moieties (9-atom spacers), which generates a polyethylene glycol-like chain. The egl linker avoids potential problem of unwanted interactions of the adenosine linkers described above for bipartite DNA and RNA ASOs. In addition, a polylysine sequence was added, as this motif (8 Lys on the C-terminus, 1 Lys on the N-terminus) is reported to greatly enhance PNA permeability in cells and tissue distribution* in vivo *[48]. Also, PNA binds much more strongly to RNA than does a corresponding DNA or RNA [49]; therefore, the designed length of the two complementary PNA regions (in green in Figure 5(b)) ranges from 4 to 9 nucleobases on either side. EMSA demonstrated that 5 nucleobases on either side are sufficient for effective binding, substantially shorter (by 2–4 bases total) than what is required for bipartite DNA or RNA ASO binding.

### 2.4. Toward Small Molecule-Antisense Conjugates [50]

While MTX is a small-molecule drug with good physicochemical and pharmacokinetic properties, major problems with pursuing MTX to target tau mRNA include its toxicity and lack of specificity. MTX is an anticancer drug that inhibits DNA topoisomerase [51], and MTX binds to other RNA hairpin structures besides that of tau (unpublished results). Moreover, even with the NMR structure of MTX bound to the stem-loop [39], structure-based design of MTX analogs to enhance potency and selectivity will not likely lead to compounds with dramatically improved properties, as similar approaches over the years with other small-molecule RNA ligands (e.g., neomycin) have not generated dramatic improvements in potency and, more importantly, specificity [52]. In contrast to small molecule ligands, the bipartite ASOs described above have excellent specificity and alter tau splicing in cells, but these agents do not have good drug-like properties. We have begun to combine these approaches, to generate MTX-ASO conjugates, in order to eliminate the toxicity of MTX, increase specificity, and demonstrate that such “molecular clasps” can effectively bind the tau stem-loop and flanking sequences and shift splicing away from the deleterious 4R isoforms in cells.

**Figure 6:**
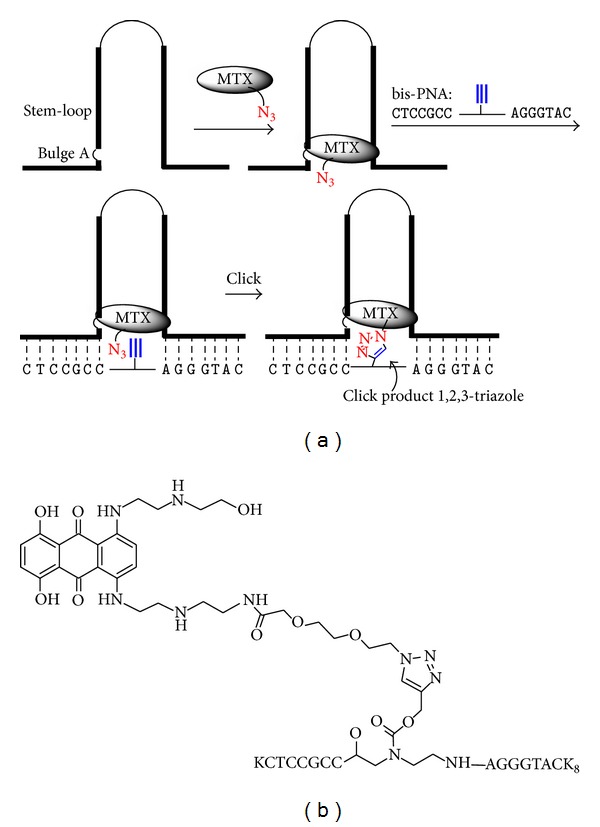
(a) Schematic of template-directed combinatorial strategy to identify MTX-PNA conjugates. MTX analogs containing a linker-azide combined bipartite PNA with a linker-alkyne in the presence of tau RNA oligonucleotide representing the stem-loop and flanking sequences. MTX and PNA analogs that bind well, with the proper distance and orientation between azide and alkyne, undergo cyclization to a 1,2,3-triazole. (b) MTX conjugated to bipartite PNA identified through template-directed combinatorial chemistry.

We selected PNAs as the initial class of antisense molecules to carry out conjugation to MTX, as these bipartite ASOs bound to tau RNA even when shortened to 5-6 nucleobases on either side. To identify PNA-MTX conjugates capable of binding the tau mRNA, we carried out a template-directed synthesis (Figure 6(a)). A 40-residue RNA oligo was used as a template to bring together PNA and MTX fragments with reactive functional groups. Specifically, we employed the Huisgen 1,3-dipolar cycloaddition reaction (often referred to as “click chemistry”), which involves triazole ring formation between azide and alkyne functionalities. This type of reaction is highly specific and has even been accomplished in living mice [53–55]. Previous reports have described template-directed reactions using enzymes [56–60] or nucleic acids [61–64] as templates.

Concerning the general design of MTX fragments, we had already determined that the chains on the anthraquinone ring system can be extended with retention of tau stem-loop binding (e.g.,** 2**, Figure 3) [40]. Two different length linkers, of 8 and 17 atoms and with azide groups on the ends, on MTX were synthesized to maximize the chance of coupling, although both were expected to be capable based on the MTX-RNA structural model determined by NMR (the binding site is close to the base of the stem and therefore near the flanking sequences where the bipartite antisense probes bind). We also synthesized linker-extended forms of tri- and tetra-substituted** 3** and** 4** (Figure 3), which also effectively bound to the tau stem-loop structure [40]. For PNA, we chose several different linkers joining the two antisense “arms” which varied in the position of an alkyne substituent for reaction with the azide on the MTX fragment. Altogether, six different MTX analogues and ten different PNA oligos were combined, for a total of 60 different reactions, in the presence of the tau-based RNA oligo. Only one of these combinations led to successful template-directed coupling, providing compound** 16** (Figure 6(b)) as the first candidate molecular clasp. This compound was capable of binding the tau-based oligonucleotide and inhibited tau splicing* in vitro* better than the bipartite PNA alone. However, the compound still proved too cytotoxic to demonstrate effects on tau splicing in cells. Means to improve this prototype compound include replacing MTX with an RNA stem-loop binding compound that is nontoxic.

### 2.5. Regulation of Tau mRNA via Its 3′-Untranslated Region [65]

As another means of targeting tau mRNA, we considered its 3′-untranslated region (3′-UTR). In general, 3′-UTR has a variety of functions in regulating gene expression, such as 3′-end processing, translational regulation, stability, and subcellular localization of the transcript [66]. These regulatory roles are mediated by the interaction of* cis*-elements in the 3′-UTR and* trans*-factors, which include proteins and microRNAs (miRNAs), in the cell [67]. An additional level of regulation results from alternative polyadenylation, which can occur in transcripts that contain two or more polyadenylation signals. Transcripts that undergo alternative polyadenylation express alternative 3′-UTR isoforms, which can be differentially regulated by either the inclusion or exclusion of regulatory* cis*-elements [68]. The human tau 3′-UTR, as well as that of rodents, contains two polyadenylation signals in tandem and can undergo alternative polyadenylation to produce transcripts of approximately 2 or 6 kb [69].

We sought to investigate the role of the human tau 3′-UTR in regulating tau expression with the hope that a deeper understanding of these regulatory mechanisms may lead to the identification of novel therapeutic approaches to treat AD and other tauopathies [65]. First, the exact sequences of the short and long tau transcripts resulting from alternative polyadenylation were determined by 3′-RACE (rapid amplification of cDNA ends) and found to be 254 and 4163 nucleotides long, respectively. These data were used to clone the wild-type and short tau 3′-UTR sequences into a luciferase reporter construct. A similar construct that expresses only the long tau 3′-UTR was made by mutating the proximal tau polyadenylation signal in the wild-type tau 3′-UTR constructs so that cleavage as well as polyadenylation does not occur at this site. Using these reporter constructs, we found that the expression of the short isoform construct is significantly higher than that of the long isoform construct in both SH-SY5Y and M17D neuroblastoma cell lines at the level of both protein and mRNA. In general, the wild-type tau 3′-UTR reporter, which contains both polyadenylation signals, had intermediate expression levels. These results suggest that the long form of the tau 3′-UTR contains* cis*-elements not found in the short form that downregulate expression.

Since miRNAs often bind 3′-UTR and regulate gene expression, we considered whether one or more miRNAs might contribute to the control of tau expression. Using a candidate approach, we found that miR-34a could specifically reduce reporter expression at both the mRNA and protein levels, as mutation of the miR-34a binding seed region in the tau 3′-UTR prevented the knockdown. Importantly, miR-34a could specifically reduce endogenous tau expression at both the mRNA and protein levels in M17D cells. To determine the ability of endogenous miRNAs to regulate endogenous tau expression, we cotransfected locked nucleic acid (LNA) miRNA inhibitors of miR-34a, -34b, and -34c into M17D cells. We used a combination of LNA inhibitors of all miR-34 family members to prevent possible compensation by other family members. Inhibition of endogenous miR-34 family members led to a moderate increase in endogenous tau protein expression, suggesting that these miRNAs do indeed repress tau levels. In addition to the miR-34a binding site on the tau 3′-UTR, other regions containing relevant* cis*-acting elements were identified by inserting defined fragments into the luciferase reporter vector. Thus, miR-34a and perhaps other miRNAs could in principle be used therapeutically to reduce tau levels.

## 3. BACE1 mRNA

### 3.1. Antisense Strategies to Shift Alternative mRNA Splicing of BACE1 [70]

**Figure 7:**
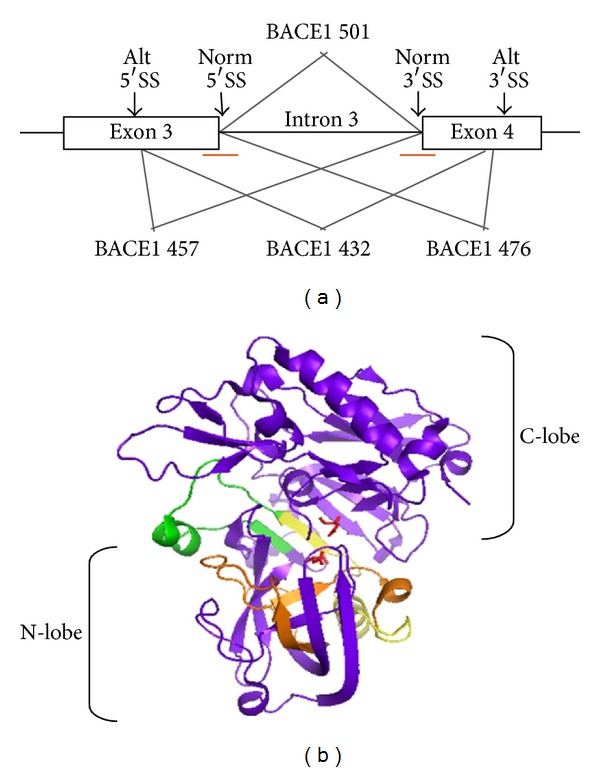
BACE1 alternative splicing and targeting by antisense oligonucleotides. (a) Alternative splice sites in exons 3 and 4 of BACE1 mRNA. The amino acid length of each isoform upon translation to protein is indicated. Red lines indicate antisense oligonucleotides targeted to the normal 5′ and 3′ splice sites. (b) Structure of BACE1 showing the N- and C-terminal lobes, with the active site at the interface. Colored regions are parts of the full-length BACE1 enzyme that would be deleted in alternative splice isoforms. Note that these sites are in and around the active site and would be expected to affect the folding and final conformation of the enzyme.

The pre-mRNA of BACE1 is alternatively spliced through the use of alternative splice sites in exons 3 and 4 (see Figure 7(a)). Normal splicing of BACE1 results in production of the full-length 501-amino acid active protein (BACE1 501), while alternative splicing of BACE1 results in production of isoforms BACE1 476 (through use of the alternative exon 4 splice site), BACE1 457 (through use of the alternative exon 3 splice site), or BACE1 432 (through use of both alternative sites in exons 3 and 4). We carefully quantified these alternatively spliced BACE1 transcripts in brain and pancreas and identified a novel, albeit very minor, splice variant of BACE1 as well (BACE1 455, with exon 4 completely skipped) [70]. We found that the cellular environment can affect BACE1 alternative splicing, as different cell types can give different proportions of these splice variants. Contrary to a previous report [71], however, we did not find differences in BACE1 splice variants in different brain regions.

Molecular modeling of the BACE1 protein (for which crystal structures are available [72]) suggested that the alternative splice isoforms would have a disrupted structure, as the deleted regions appear to be critical parts of the core structure near the active site [70] (Figure 7(b)). Consistent with this hypothesis, we found that isolated BACE1 proteins translated from alternatively spliced transcripts have essentially no *β*-secretase activity. While previous studies had examined A*β* production in cells transfected with BACE1 variants, isolation of these variants was not carried out, leading to inconsistent results [73–75]. Furthermore, promotion of BACE1 alternative splicing using ASOs directed to the normal splice sites (red lines, Figure 7(a)) substantially reduced A*β* production in cells. Total BACE1 mRNA levels were not altered by treatment with these ASOs. These findings illustrate the importance of BACE1 alternative splicing in affecting the level of A*β* produced in cells and suggest that targeting regulation of BACE1 alternative splicing is a potential therapeutic strategy for reducing *β*-secretase activity.

### 3.2. Role of a G-Quadruplex Structure in BACE1 mRNA Splicing [76]

**Figure 8:**
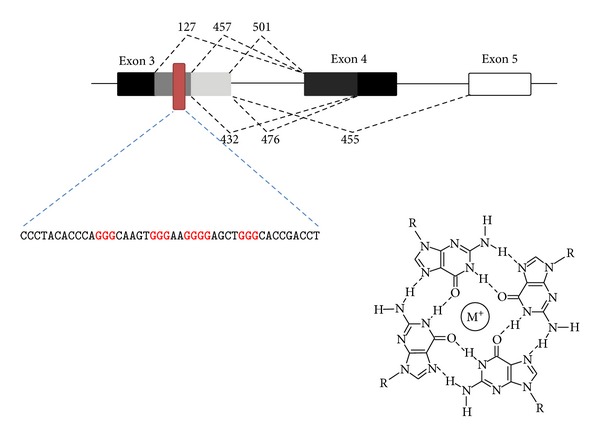
Site of four successive GGG motifs in exon 3 of BACE1 mRNA and alternative splice sites. These motifs fold into a G-quadruplex structure, with stacked sets of four planar guanine rings stabilized by Hoogsten base pairing and a K+ ion.

We recently identified another* cis*-element within the BACE1 mRNA, one that putatively folds up into a G-quadruplex, in which G-rich stretches form four-stranded structures consisting of square arrangements of guanines stabilized by Hoogsteen hydrogen bonding and K+ chelation (Figure 8) [77]. Four proximal GGG motifs are found within exon 3, near the alternative 5′ splice site leading to BACE1 457 and another alternative site leading to a form with a premature stop codon after 127 amino acids [75] (BACE1 127) (Figure 8). Mutation of any of these GGG motifs to GAG (the GGGG motif was mutated to GAAG) within a BACE1 exon 3–5 minigene construct led to substantial increases in BACE1 457 and 432 splicing by qRT-PCR in HEK293 cells [76], consistent with these motifs being part of a* cis*-element that represses use of this alternative splice site. Little or no effect was seen on levels of BACE 476 (alternative exon 4 splice site) or 455 (exon 4 skipped) splice isoforms. For most mutants, a significant decrease in full-length BACE1 501 splice isoform was also seen. Mutations in other minigene systems further supported the importance of these four proximal GGG motifs in regulating the use of the alternative 5′ splice site in exon 3. For example, placement of the putative BACE1 G-quadruplex motif into a *β*-globin minigene system near an alternative 5′ splice site is sufficient to cause exon skipping.

Nuclease protection assays, nuclear magnetic resonance, and circular dichroism spectroscopy revealed that this 4 x GGG sequence indeed folds into a G-quadruplex structure stabilized by K+ ions [76]. Affinity pull-down using biotinylated RNA of the BACE1 4 x GGG sequence led to identification of several proteins capable of binding to the G-rich sequence, and one of these, heterogeneous nuclear ribonucleoprotein H (hnRNP H), was found to regulate BACE1 exon 3 alternative splicing and in a manner dependent on the G-rich sequence. Knockdown of hnRNP H led to a decrease in the full-length BACE1 mRNA isoform as well as a decrease in A*β* production from APP, suggesting new possibilities for therapeutic approaches to AD.

## 4. Outlook

The string of clinical failures of agents targeting proteins involved in A*β* production as well as the inability to advance agents targeting tau or tau-phosphorylating kinases argues for the pursuit of alternative strategies for AD therapeutics. Herein, we have surveyed our recent work toward targeting mRNA relevant to AD and FTLD-tau, specifically tau and BACE1 mRNA.

For tau mRNA, we have validated a stem-loop structure that regulates exon 10 splicing, thereby controlling the critical 4R/3R ratio implicated in FTLD-tau pathogenesis. With this validated target, we screened for and identified a small molecule, MTX, that binds to and stabilizes the stem-loop, the opposite effect of FTLD-tau-causing mutations in this region. We also designed bipartite ASOs that bind the regions that flank this stem-loop and showed that these can shift tau splicing away from 4R tau and toward 3R tau. Attempts to combine MTX and bipartite ASOs to form tripartite “molecular clasps” were successful, although further modifications are needed to decrease cytotoxicity. We have also systematically studied the tau mRNA 3′-UTR, identified regions within the 3′-UTR that are critical to regulating tau mRNA and protein levels, and discovered that miR-34a reduces tau.

For BACE1 mRNA, we have validated isoforms resulting from alternative splicing with exons 3 and 4 and demonstrated that the proteins encoded by these alternative BACE1 mRNA splice isoforms have little or no proteolytic activity for producing A*β*. With this knowledge, we tested ASOs targeting the normal splice sites leading to enzymatically active, full-length BACE1 and found that these ASOs could effectively shift splicing toward the inactive alternative isoforms and reduce A*β* production. We also discovered a G-quadruplex structure within exon 3 of BACE1 mRNA that can regulate use of alternative splice sites, at least in part by recruitment of hnRNP H. Perhaps agents targeting the G-quadruplex or hnRNA H could likewise shift BACE1 mRNA splicing and lower A*β* production.

Taken together, these results provide proof of principle that targeting tau or BACE1 mRNA for potentially therapeutic ends is possible. However, much further work will be needed before this early validation can be translated into practical therapeutics for AD and FTLD-tau. Further details are needed on the mechanisms by which these mRNAs are regulated. For instance, with tau, little is known about trans-factors that interact with the 3′-UTR. Although we have discovered a role for miR-34a, other miRNAs may also contribute and perhaps some much more substantially than miR-34a. Protein trans-factors are also likely involved, some of which may be more druggable than the tau mRNA 3′-UTR itself. For BACE1, other regulating proteins besides hnRNP H are likely involved and could be discovered through affinity isolation of other regions of the BACE1 mRNA besides the G-quadruplex. For both tau and BACE1 mRNA, other structural motifs besides the stem-loop (tau) and G-quadruplex (BACE1) are also likely to be present, and a systematic search for such structures would be warranted.

Such basic understanding of tau and BACE1 mRNA structure and function may reveal new strategies for targeting these mRNAs for therapeutic purposes. Toward that end, however, there is a great need to investigate new means of discovering agents that bind RNA structures. This area of chemical design is still in its infancy, and there are few if any agents that target specific RNA structures. While oligonucleotides such as ASOs, siRNAs, and miRNAs can provide such specificity, these agents have poor drug-like properties. Thus, another important avenue of investigation is the development of practical delivery strategies of designed oligonucleotides to the brain. This is a daunting challenge, but efforts must be made given the strong therapeutic potential of oligonucleotides and the lack of any effective therapeutics for the devastating and widespread problems of AD and FTLD-tau.
